# BK in Double-Membrane Organelles: A Biophysical, Pharmacological, and Functional Survey

**DOI:** 10.3389/fphys.2021.761474

**Published:** 2021-10-26

**Authors:** Naileth González-Sanabria, Felipe Echeverría, Ignacio Segura, Rosangelina Alvarado-Sánchez, Ramon Latorre

**Affiliations:** Facultad de Ciencias, Centro Interdisciplinario de Neurociencia de Valparaíso, Universidad de Valparaíso, Valparaíso, Chile

**Keywords:** BK channel, mitoBK, nBK, BK pharmacology, mitochondria, nucleus

## Abstract

In the 1970s, calcium-activated potassium currents were recorded for the first time. In 10years, this Ca^2+^-activated potassium channel was identified in rat skeletal muscle, chromaffin cells and characterized in skeletal muscle membranes reconstituted in lipid bilayers. This calcium- and voltage-activated potassium channel, dubbed BK for “Big K” due to its large ionic conductance between 130 and 300 pS in symmetric K^+^. The BK channel is a tetramer where the pore-forming α subunit contains seven transmembrane segments. It has a modular architecture containing a pore domain with a highly potassium-selective filter, a voltage-sensor domain and two intracellular Ca^2+^ binding sites in the C-terminus. BK is found in the plasma membrane of different cell types, the inner mitochondrial membrane (mitoBK) and the nuclear envelope’s outer membrane (nBK). Like BK channels in the plasma membrane (pmBK), the open probability of mitoBK and nBK channels are regulated by Ca^2+^ and voltage and modulated by auxiliary subunits. BK channels share common pharmacology to toxins such as iberiotoxin, charybdotoxin, paxilline, and agonists of the benzimidazole family. However, the precise role of mitoBK and nBK remains largely unknown. To date, mitoBK has been reported to play a role in protecting the heart from ischemic injury. At the same time, pharmacology suggests that nBK has a role in regulating nuclear Ca^2+^, membrane potential and expression of eNOS. Here, we will discuss at the biophysical level the properties and differences of mitoBK and nBK compared to those of pmBK and their pharmacology and function.

## Introduction

In the 1980s, the calcium-activated potassium channel was identified for the first time in rat skeletal muscle (Pallotta et al., 1981), chromaffin cells (Marty, 1981), and skeletal muscle membranes incorporated in lipid bilayers (Latorre et al., 1982). The BK channel has a large ionic conductance (~250 pS in symmetrical 100mMK^+^) and an exceptional K^+^ selectivity, hallmarks that established the name of BK “big K^+^” (Marty, 1983) or MaxiK (Latorre and Miller, 1983).

The BK channel is regulated by intracellular Ca^2+^ concentration and the membrane potential difference (Marty, 1981; Pallotta et al., 1981; Latorre et al., 1982). Both properties allow it to work in a wide range of membrane potentials and intracellular Ca^2+^ concentrations. BK has been described in different cell types and organelles (Singh et al., 2012; Li and Gao, 2016). Given the ubiquitous distribution of the BK channel and the variety of physiological roles in which it is involved, it is reasonable to think that channel alteration may have severe consequences in various channelopathies.

The BK channel is a member of the super family of K^+^ voltage-dependent channels (Kv) encoded by the KCNMA1 gene (Latorre et al., 2010). BK is a homotetramer, and each of the α subunits consists of seven transmembrane segments (S0–S6). Segments S0–S4 constitute the voltage sensor domain (VSD) and segments S5–S6 the pore domain (PD). The C-terminal region located on the intracellular side contains two K^+^-conductance regulators (RCK1 and RCK2) where the Ca^2+^- binding sites reside (Yuan et al., 2010; Hite et al., 2017; Tao et al., 2017).

Although our knowledge of ion channel biophysics and pharmacology has increased enormously in recent years, the biophysical properties and pharmacology of different variants of BK that are expressed in organelles, especially in double-membrane organelles, need more detailed studies. However, despite the obvious structural and functional importance of the nucleus in gene expression and regulation, the role of nuclear BK channels (nBK) in intracellular signaling pathways is not fully understood (Li et al., 2014; Selezneva et al., 2021).

**Figure 1:**
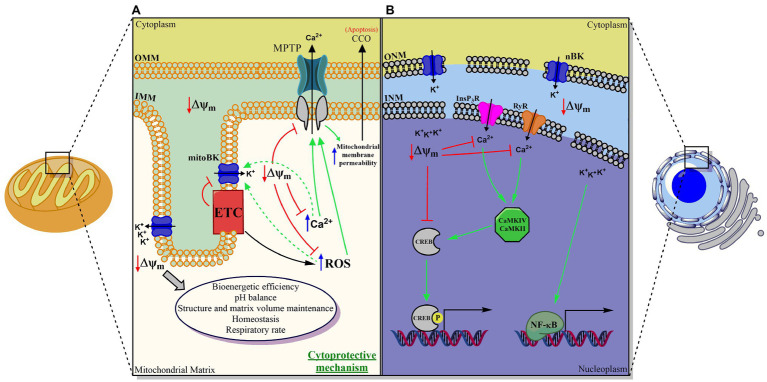
Proposed scheme for the functional role of the BK channel in double-membrane organelles. **(A)** Mitochondria, **(B)** Nucleus. In both organelles, BK plays a fundamental role in maintaining the transmembrane potential and in regulating the movement of the calcium ion between the cytosol and the organelle, and between the lumen and the internal membrane of the same. MitoBK is involved in mitochondrial function, structure, homeostasis, and volume, as well as pH control, bioenergetic efficiency, respiratory rate (through a structural and functional assembly with the chain electron transporter (ETC)), in the closure of the mitochondrial permeability transition pore (MPTP) and with it, indirectly, in the release of cytochrome C oxidase (CCO). Thus, it would ultimately be involved in apoptosis and death cells. nBK is involved in pCREB-dependent gene regulation (in principle regulated by Calmodulin-dependent kinases (CaMKIV and CaMKII)) and NF-kB, mechanisms by which, to date, nBK has been associated with neuronal survival and response inflammatory mediated by macrophages, respectively.

To understand the nBK functional importance, we need to comprehend Ca^2+^ storage and signaling in nuclei and how the nuclear envelope (NE) is involved. First of all, the NE consists of two concentric lipid bilayers. The outer nuclear membrane (ONM), which is continuous with the endoplasmic reticulum membrane, and the inner nuclear membrane (INM). Within the NE, InsP3R, which is a Ca^2+^ permeable channel, can be found in both the ONM and the INM (Leite et al., 2003). There is the perinuclear space located between the ONM and the INM, which is a crucial source of Ca^2+^ that can be released into the nucleoplasm not only through InsP3R, but also using of ryanodine receptors (RyR; see Figure 1B; Zahradníková and Mészáros, 1998). A critical effect of nuclear Ca^2+^ increase is phosphorylation and activation of cAMP response element-binding protein (CREB), which regulates many genes of different cell types, such as neurons that elicits transcription of genes that promotes neuronal survival (Papadia et al., 2005).

Multiple investigations suggest that both mitoBK and nBK have the same structure as pmBK, and they share biophysical and pharmacological properties (Singh et al., 2012; Balderas et al., 2015; Li and Gao, 2016). Although it has been assumed that the same pharmacology for pmBK applies to BK channels contained in organelle membranes, some examples show unexpected effects of BK blocking agents. For instance, charybdotoxin (ChTX), a high-affinity BK blocker, could not block a BK like-channel characterized in mitochondria (Singh et al., 2012), as similarly reported by Meera et al. (2000) for coexpression of α with the β4 subunit, where β4 renders the BK channel insensitive to ChTX (Meera et al., 2000; Torres et al., 2014).

This review will summarize all the biophysical, pharmacological, and functional information that exists to date on the mitoBK and nBK channels, with a comparative perspective over pmBK features.

## MitoBK and nBK Localization in Organelles and Tissues

Mitochondria are crucial for cell survival, and vital cellular processes occur in this organelle. Therefore, understanding the different ion channels interplay in the mitochondrial membranes could be helpful in the modulation of diverse mitochondrial-related molecular mechanisms and thus cellular processes such as the apoptosis or hypoxia response (Wallace, 1999; Kim et al., 2006; Papandreou et al., 2006). Impairment of the mitochondrial membrane potential leads to the release of cytochrome c from the mitochondrial membrane, an essential process for the induction of cell death. Therefore, the study of ion channels in the mitochondrial membranes became an exciting subject at the end of the 20th century (Borecký et al., 1997; Siemen et al., 1999).

Important diseases that include mitochondria failures may well involve the presence of potassium channels. In the search for anti-ischemic drugs, Xu et al. (2002) were the first to find clear electrophysiological evidence aiming to an isoform of the BK channel within the cardiac myocyte inner mitochondrial membrane (IMM) of guinea pig hearts. This channel carried a large portion of the K^+^ uniport activity and led to the finding of the ischemic insult-protecting role of the mitoBK. Likewise, the mitoBK channel was also found in rat hearts, specifically in cardiac ventricular myocytes (Ohya et al., 2005).

Using western-blot, immunocytochemistry, and inmuno-gold electron microscopy, Douglas et al. (2006) showed that mitoBK is present in the rat’s brain mitochondria. Considering that ischemic-brain injury-related hypoxia has substantial effects on neuron metabolism and survival, it is remarkable that hypoxic conditions activated mitoBK from rat brain astrocytes (Cheng et al., 2008). Similarly, it has been demonstrated that hypoxia activates the BK channels present in mitoplast derived from the human glioma LN-229 cells (Gu et al., 2007), and the same effect was also found in the mitoplast of liver mitochondria (Cheng et al., 2008). In addition, submitochondrial particles extracted from rat hippocampal neurons were reconstituted into lipid bilayer membranes. Thus, electrophysiological recordings and confocal immunohistochemical images confirmed the presence of the mitoBK channel, including its accessory β4 subunit with an apparent molecular weight of ~26kDa (Skalska et al., 2009). Recalling here that β4 is present in the plasma membrane of neurons in the brain (Weiger et al., 2000), it was demonstrated, using western blot analysis, that β4 and β2 subunits are present in brain homogenates and mitochondrial fractions (Piwonska et al., 2008). Additionally, the β2 subunit was also identified in human epithelial cell line mitoplasts; however, the idea that it does not form a complex with mitoBK is disputed (Bednarczyk et al., 2013a) since it does not show the time-dependent inactivation that this subunit confers on membrane BK channels (Wallner et al., 1999; Benzinger et al., 2006; Torres et al., 2014).

Later on, more studies confirmed mitoBK presence in cardiomyocytes (Singh et al., 2013; Soltysinska et al., 2014; Frankenreiter et al., 2017) and in brain tissues (Fahanik-Babaei et al., 2011a,b; Singh et al., 2012; Augustynek et al., 2018).

Recently, mitoBK channels were well-described in human glioma cell lines (Gu et al., 2007; Walewska et al., 2018; Gałecka et al., 2021), supporting the data of Siemen et al. (1999) in their first attempt to obtain mitoBK currents from human glioma cells. Further, mitoBK was characterized in other human cell lines from the endothelium, fibroblast, and glioblastoma to detect the expression of different splice variants and the co-assembly with different types of auxiliary β subunits that may affect the complexity of the mitoBK channel gating (Wawrzkiewicz-Jałowiecka et al., 2021). β4 expression with mitoBK has been found in H9c2 cell line derived from heart (Fretwell and Dickenson, 2009), in human astrocytoma cells U-87 MG rat skeletal muscle (Bednarczyk et al., 2013b), and in thalamus and brainstem (Piwonska et al., 2008). On the other hand, the β2 subunit has been reported in the human endothelium EA.hy926 cell line (Bednarczyk et al., 2013a) and in rat brain (Piwonska et al., 2008), while β3 is highly expressed in human fibroblasts, in which β2 and β4 subunits were also found (Kicinska et al., 2016).

Nonmammalian cells have been a point of interest for the search of mitochondrial potassium channels. More precisely, a mitoBK channel-like protein in potato tuber cells was characterized by obtaining similar properties as the pmBK channel with a remarkably exception of the single-channel conductance of about ~600 pS (Koszela-Piotrowska et al., 2009). The functional and pharmacological features of the mitoBK channel were also characterized in the IMM of *Dictyostelium discoideum* a unicellular ameboid protozoon that forms multicellular structures. The mitoBK of this protozoon was characterized using electrophysiological measurements, immunoblotting, and functional measurements of oxygen uptake and Δψ changes (Laskowski et al., 2015).

Like the mitochondria and other membrane-bound organelles, as already mentioned, the nuclear envelope (NE) is also made up of two separated membranes: the inner nuclear membrane (INM), which interacts with the nuclear skeleton, and the outer nuclear membrane (ONM), which is continuous with the endoplasmic reticulum (Fedorenko et al., 2010; Singh et al., 2012). The presence of a potassium channel activated by Ca^2+^ and voltage was reported in the outer nuclear membrane of pancreatic acinar cells, with a single-channel conductance between 180 and 200 pS (Maruyama et al., 1995). Later, the presence of nBK channels was also confirmed in isolated nuclei from brain microvessel endothelial cells (Gobeil et al., 2002).

Further, the nBK channel was found in the nuclear membrane of mouse hippocampal neurons, and immunohistochemical assays clarified that BK is not present in the nucleus of BK-knockout mouse neurons (*KCNMA1* −/−; Li et al., 2014). Single-channel recordings in isolated nuclei from hippocampal neurons further confirmed the presence of nBK channels (Li et al., 2014). The single-channel conductance obtained was similar to that reported for the pmBK channel present in neurons (Salkoff et al., 2006).

Like neuronal pmBK channels, nBKs form complexes with β4 helper subunits (Shruti et al., 2012; Li et al., 2014). More recently, Chen et al. reported the presence of the nBK channel in Ampullae of Lorenzini cells (an electroreceptor organ of cartilaginous fish) from *in situ* assays using confocal microscopy and immunostaining (Chen et al., 2020).

Altogether, the data suggest that the mitoBK and nBK channels have the same mammalian-tissue localization as the pmBK channel (Dworetzky et al., 1994; Knaus et al., 1996; Poulsen et al., 2009; Chen et al., 2010).

## Biophysical Properties and Differences to pmBK

### Mitochondrial BK Biophysical Properties

mitoBK single-channel recordings were reported for the first time in mitoplasts from glioma human cell line LN229 mitochondria (Siemen et al., 1999). Since then, patch-clamp experiments from mitoBK have been carried out not only using mitoblasts (Xu et al., 2002; Ohya et al., 2005; Gu et al., 2007; Cheng et al., 2008, 2011; Bednarczyk et al., 2013a,b; Soltysinska et al., 2014; Frankenreiter et al., 2017; Walewska et al., 2018; Balderas et al., 2019) but also lipid bilayers (Skalska et al., 2009; Fahanik-Babaei et al., 2011a,b). Overall, it has been found that mitoBK shares similar behavior to pmBK (see Table 1), with a unitary conductance (*γ*) around 282±23 pS in multiple K^+^ conditions, a voltage-dependent open probability (*P_o_*) (Siemen et al., 1999; Bednarczyk et al., 2013b) which shows a leftward shift in the P_o_ – Voltage curves when increasing Ca^2+^ levels (Xu et al., 2002; Balderas et al., 2019) and sensitivity to negative hydrostatic pressure (Walewska et al., 2018). Interestingly, mitoBK *P_o_* increases under hypoxic conditions (Gu et al., 2007; Cheng et al., 2008). BK localization in mitochondria is a result of VEDEC splice variant from *KCNMA1* gene, which has been described with the aforementioned properties (Singh et al., 2013; Gałecka et al., 2021).

**Table 1:** Biophysics and Pharmacology of mitoBK and nBK.

Tissue/organism	Methodology	Unitary conductance (pS) and conditions	Biophysical parameters	Pharmacology	References
Potato tuber (mitoBK)	Lipid bilayer	615±12 – gradient cis/trans 50/450mMK^+^	*V_r_* =+34mV, *P_0_* =0.49 (0 Ca^2+^; 0mV)/0.84 (300μM Ca^2+^; 0mV)	NS1619 Iberiotoxin	Koszela-Piotrowska et al., 2009
Rat brain (mitoBK)	Lipid bilayer	~565 – gradient cis/trans 200/50mMK^+^	*V_r_* =−30mV	Iberiotoxin charybdotoxin	Fahanik-Babaei et al., 2011b; Singh et al., 2012
Drosophila melanogaster (mitoBK)	Single channel in mitoplast	382±8 – symmetric 150mMK^+^	N/A	NS1619 Paxilline	Gururaja et al., 2019
Guinea pig cardiomyocytes (mitoBK)	Single channel in mitoplast	307±4.6 – symmetric 150mMK^+^	N/A	NS1619 NS11021 Paxilline	Xu et al., 2002; Aon et al., 2010
Rat cardiomyocites (mitoBK)	Single channel in mitoplast	303±19 – symmetric 140mMK^+^	V_1/2_ =−55mV (12μM Ca^2+^)	diCl-DHAA paxilline	Sakamoto et al., 2008; Balderas et al., 2019
Rat astrocytes (mitoBK)	Single channel in mitoplast	296±18 – symmetric 150mMK^+^	N/A	CGS7181 CGS7184 paxilline charybdotoxin	Cheng et al., 2008; Augustynek et al., 2018
Human glioma (mitoBK)	Single channel in mitoplast	295±18 – symmetric 150mMK^+^	*V_r_* =+70mV (150mM Na^+^), EC_50_(Ca^2+^)=900nM (+60mV)/6.9μM (−20mV)	Charybdotoxin	Siemen et al., 1999
Rat non-neoplastic astrocytes (mitoBK)	Single channel in mitoplast	~290 – symmetric 140mMK^+^	N/A	Iberiotoxin	Cheng et al., 2011
HEK293T (DEC splice variant; mitoBK)	Single channel in mitoplast	290±3 – symmetric 150mMK^+^	V_1/2_ =+19.2mV (100μM Ca^2+^), *t_0_* =10.2ms (+60mV)	Paxilline	Gałecka et al., 2021
Human glioma (mitoBK)	Single channel in mitoplast	276±9 – symmetric 150mMK^+^	*V_r_* =+9.3±2.4mV; O_2_: 21.1±1.2nmol/ml	Charybdotoxin	Gu et al., 2007
Rat ventricular myocytes (mitoBK)	Single channel in mitoplast	~270 – symmetric 140mMK^+^	N/A	17B-estradiol paxilline	Ohya et al., 2005
Human endothelium (mitoBK)	Single channel in mitoplast	270±10 – symmetric 150mMK^+^	*t_0_* =0ms (−60mV)/~70ms (+60mV)	NS1619 NS11021 paxilline iberiotoxin	Bednarczyk et al., 2013a
Rat brain (mitoBK)	Lipid bilayer	265±5 – gradient cis/trans 50/450mMK^+^	*V_r_* =+50mV, P_o_ (+70mV)=0.5 (0 Ca^2+^)/0.77 (300μM Ca^2+^)	NS1619 iberiotoxin charybdotoxin	Skalska et al., 2009
Human glioma (mitoBK)	Single channel in mitoplast	262±12 – symmetric 150mMK^+^	N/A	Charybdotoxin	Walewska et al., 2018
*Dictyostelium discoideum* (mitoBK)	Lipid bilayer	258±12 – gradient cis/trans 50/150mMK^+^	*V_r_* =+27.6±0.5mV, *P_0_* =0.14 (1μM Ca^2+^; 0mV)/0.48 (100μM Ca^2+^; 0mV)	NS1619 NS11021 paxilline iberiotoxin	Laskowski et al., 2015
Rat hippocampal neurons (nBK)	Single channel in nuclear envelope	217 pS – symmetric 135mMK^+^	*V_r_* =0mV, *P_0_* =0.3 (5μM Ca^2+^)/0.78 (10μM Ca^2+^)	Paxilline	Li et al., 2014
Rat brain (mitoBK)	Lipid bilayer	~211 – gradient cis/trans 200/50mMK^+^	*V_r_* =−30mV	Iberiotoxin	Fahanik-Babaei et al., 2011a; Singh et al., 2012
Rat pancreas (nBK)	Single channel in nuclear envelope	200±25 pS – symmetric 148mMK^+^	*V_r_* =0mV	N/A	Maruyama et al., 1995
Mice cardiomyocytes (mitoBK)	Single channel in mitoplast	~190 – internal/external=130:10mMK^+^	*t_0_* =9.23ms, *P_0_* =0.79 (+80mV)	NS11021 Paxilline	Soltysinska et al., 2014
Mice cardiomyocytes (mitoBK)	Single channel in mitoplast	~145 – symmetric 150mMK^+^	*P_0_* =0.28 (1μM Ca^2+^); 0.54 (100μM Ca^2+^)	NS11021 Paxilline	Frankenreiter et al., 2017

### Nuclear BK Biophysical Properties

The first identified calcium- and voltage-activated potassium channel (nBK) in nucleus was characterized in rat pancreatic acinar cells at the single-channel level using the patch-clamp technique (Maruyama et al., 1995). Although there was no pharmacology approaches or microscope imaging, this study was pioneer in the search of BK channels in other intracellular organelles membranes. It has been found that nBK shares similar behavior to pmBK (Singh et al., 2012).

Almost 20years later, functional nBK channels were described using a set of different techniques, including immunoelectron microscopy and confocal fluorescence. Most importantly, single-channel recordings in isolated nuclei showed a P_o_ of 0.3 using 5μM Ca^2+^, increasing to ~0.8 at 10 uM Ca^2+^, indicating the presence of a Ca^2+^−activated channel with a *γ*=217 pS (Table 1; Li et al., 2014).

### Regulation of mito-BK and nBK by Auxiliary β Subunits

Likewise, as in pmBK, the accessory β1 subunit can assemble with the α subunit of mitoBK and nBK (Ohya et al., 2005; Li et al., 2014; Balderas et al., 2020). This subunit modify the pharmacological characteristics and gating of the channel. Recent findings have revealed the presence of mitoBK channels formed by the β1/α complex in mammalian myocyte mitochondria (Ohya et al., 2005; Testai et al., 2017; Balderas et al., 2020). β1 regulates expression and targets mitoBK to the IMM and changes the channel voltage sensitivity (Balderas et al., 2020). These results could explain how is possible to activate mitoBK in the mitochondrial environment (ΔΨ~−200mV, [Ca^2+^]_mit_≈200μM). Under these conditions, the β1/α mitoBK conductance–voltage curve is leftward shifted and the channel shows an appreciable P_o_ (Ohya et al., 2005; Bautista et al., 2009; Balderas et al., 2020). The accessory β1 subunit detected in mitoplast from rat ventricular myocytes interacts with the cytochrome C oxidase (CCO), confirming the mitoBK-β1 complex association with the respiratory electron transport chain in heart mitochondria (Ohya et al., 2005). In addition, the mitoBK-β1 complex was also found in cultured pulmonary artery smooth muscle mitochondria (Loot et al., 2012).

mitoBK was characterized in other human cell lines from the endothelium, fibroblast, and glioblastoma where multiple splice variants were found that co-assemble with different types of auxiliary β subunits that may affect the complexity of the mitoBK channel pharmacology and gating (Wawrzkiewicz-Jałowiecka et al., 2021).

At present, only β4 expression has been reported in nuclear membranes co-localizing with B-type lamin (Li et al., 2014.). However, there are many questions that still need to be answered regarding the detailed mechanism of how accessory subunits are directed and assembled in IMM and ONM, as well as the modulatory effect they exert on mitoBK and nBK. Regarding γ subunits, the association with the mitoBK-α and nBK-α is still to be addressed (González-Cota et al., 2021).

## Pharmacological Properties

### Mitochondrial BK Pharmacological Properties

The basic pharmacology properties of mitochondrial potassium channels like mitoK_ATP_, mitoBK, and mitoKv1.3 are similar to their equivalents in plasma membrane from different cell types (Szewczyk et al., 2006; Laskowski et al., 2016). Therefore, activators and inhibitors previously described for the pmBK channel can exert the same effect on mitoBK (O’Rourke, 2007; Szewczyk et al., 2010). Different reports indicate that nonspecific interactions of potassium channel modulators may occur, indicating that these compounds may influence cell and mitochondrial function regardless of their main targets (Szewczyk et al., 2010; Laskowski et al., 2016; Augustynek et al., 2018).

CGS7181(ethyl2-hydroxy-1-[[(4-methylphenyl)amino]oxo]-6-trifluoromethyl-1H-indole carboxylate) is an indole carboxylate derivative that, just as its analog CGS7184 (ethyl 1-[[(4-chlorophenyl)amino]oxo]-2-hydroxy-6-trifluoromethyl-1H-indole-3-carboxylate), activates mitoBK in single-channel recordings from astrocytoma (Augustynek et al., 2018). Using the inside-out patch-clamp configuration, they report that the open probability (NPo) increases from 0.09 in the control to 0.55 in the presence of 1μM of CGS7181. This activity was subsequently inhibited by adding 10 μM of paxilline to the bath (mitochondrial matrix; Augustynek et al., 2018). Augustynek et al. (2018) proposed that activation of mitoBK by CGS7184 induces an influx of potassium ions into the negatively charged mitochondrial matrix and promotes a light uncoupling of mitochondria. This uncoupling stimulates the activity of the mitochondrial respiratory chain to restore the potential of the mitochondrial membrane by pumping protons from the matrix into the mitochondrial intermembrane space (Augustynek et al., 2018). The agonistic effect observed in the presence of CGS7184 is dependent on potassium and charybdotoxin, indicating that the target of this compound is mitoBK (Augustynek et al., 2018).

Sakamoto demonstrated that 12,14-dichloro dehydroabietic acid (diCl-DHAA) activates mitoBK (Sakamoto et al., 2008) similarly as it activates the pmBK channel (Ohya et al., 2005). Additionally, adding 3 μM of paxilline eliminates channel opening events, allowing the authors to confirm that diCl-DHAA activates the mitoBK channel, likewise the pmBK channel (Sakamoto et al., 2008). Finally, they evaluated the protective effects of diCl-DHAA against ischemic cell death in cardiomyocytes by using the simulated ischemia procedure. diCl-DHAA has protective effects on cardiac myocytes against ischemic injury through the opening of mitoBK channels, supporting the idea that the opening of mitoBK is a novel way to protect cardiac myocytes from ischemic and reperfusion injury (Sakamoto et al., 2008).

Ohya et al. (2005) showed that 17β-estradiol could increase the mitoBK channel P_o_, activation that is inhibited by paxilline (Ohya et al., 2005). Importantly, in the presence of 17β-estradiol, cell death decreased significantly during simulated ischemia, and that this cardioprotective effect was eliminated by 3μM paxilline (Ohya et al., 2005). They concluded that this cardioprotective effect is due to the activation of mitoBK by 17β-estradiol, and since 17β-estradiol activates BK only in the presence of the β1 subunit (Valverde et al., 1999; Granados et al., 2019), this result confirms the presence of mitoBK-β1 in rat ventricular myocytes.

The benzimidazole derivatives BK activator family includes NS1619, NS004, NS1604, NS11021, and NS1643 that can activate mitoBK (Skalska et al., 2009; Szewczyk et al., 2010). NS1619 activates mitoBK at micromolar concentrations (Szewczyk et al., 2006). Moreover, the activation of mitoBK by NS1619 has a cytoprotective effect in guinea pig heart before simulated ischemia; this effect was antagonized by paxilline (Xu et al., 2002; Stowe et al., 2006; Singh et al., 2013). MitoBK activators have been reported to protect the heart against ischemic injury (Shintani et al., 2004). Furthermore, like the effect of mitoK_ATP_ activation, mitoBK opening has been implicated in preconditioning. For example, preconditioning of hearts with mitoBK activators such as NS1619 or NS11021 reduced myocardial infarction and this beneficial effect could be antagonized by co-administration with paxilline (Bentzen et al., 2009). The activation of mitoBK by NS1619 leads to cytoprotection of cardiomyocytes during ischemia/reperfusion or treatment with ouabain (Augustynek et al., 2018). However, it should considered that NS1619, like NS004, may present non-mitoBK-dependent effects in the mitochondria (Debska et al., 2003; Heinen et al., 2007b).

NS11021 exerts other protective effects by activating mitoBK channels, which are abolished in the presence of paxilline. For example, nanomolar concentrations of NS11021 improve the bioenergetic performance of the mitochondria of the heart (Aon et al., 2010; Testai et al., 2014). NS11021 also protects against ischemic injury when applied prior to ischemia or when applied immediately after reperfusion. These findings support the idea that ischemia and reperfusion-induced tissue damage can be reduced by pharmacological activation of cardiac mitoBK channels (Bentzen et al., 2009).

The following compounds are mitoBK inhibitors: charybdotoxin (Gu et al., 2007; Skalska et al., 2009; Augustynek et al., 2018), iberiotoxin (Cheng et al., 2011), and paxilline (Xu et al., 2002; Ohya et al., 2005; Sakamoto et al., 2008; Augustynek et al., 2018; Balderas et al., 2020) y Ba^2+^ (Xu et al., 2002). These compounds have been characterized previously in the pmBK having similar effects to those found in mitoBK (Szabo and Zoratti, 2014). MitoBK is inhibited by the blockers charybdotoxin, iberiotoxin, and paxilline at concentrations in the nanomolar range (O’Rourke, 2007; Singh et al., 2012). Adding 100nM of paxilline to the bath in the inside-out configuration decreases the P_o_ of the mitoBK and increases the mean close time with no effects on the mean open time. These results suggest that paxilline decreases the probability of opening by stabilizing the closed state of the channel (Balderas et al., 2020). We note here that the Lingle group who proposed that paxilline binding is state-dependent binding preferentially to the closed state of the pmBK (Zhou and Lingle, 2014).

Recently, Kravenska et al. (2020) reported in human astrocytoma cell mitoplasts that different forms of Aβ (a self-aggregating peptide) produced by cleavage of a transmembrane glycoprotein (the amyloid precursor protein involved in Alzheimer’s disease), including monomers, oligomers, and fibrils, inhibit mitoBK in a concentration-dependent manner. Five μM of Aβ fibrils, oligomers or monomers produced 80, 70, and 50% inhibition, respectively. All forms of Aβ inhibited mitoBK channel activity when applied to both sides of the membrane, indicating an indirect effect on the channel (Kravenska et al., 2020).

### Nuclear BK Pharmacological Properties

Paxilline- and iberiotoxin-specific pmBK channel inhibitors block nBK. nBK is activated by NS1619, a specific activator of pmBK. Therefore, nBK channels share similar pharmacological properties with the pmBK and mitoBK channels, targeting the same compounds (Gobeil et al., 2002; Singh et al., 2012; Li et al., 2014; Du et al., 2020).

Experiments in isolated nuclei of brain endothelial cells using NS1619 as an activator of nBK and iberiotoxin as a blocker showed that nBK is coupled to the activity of perinuclear prostaglandin receptors (EP3). Iberiotoxin abolished K^+^-dependent membrane potential changes and the expression of eNOS transcription induced by the activation of agonists of the prostanoid EP_3_-receptor, M&B 28767, while NS1619 produced Ca^2+^ transients and alterations in the perinuclear membrane potential (Gobeil et al., 2002; Singh et al., 2012).

Li et al. (2014) showed that nBKs in the nuclear envelope of hippocampal cells are functional and sensitive to pharmacological inhibition by paxilline. This compound’s blockage of nBK causes transient increases in Ca^2+^ and depolarization of the nucleoplasm relative to the perinuclear lumen, thus affecting the transcription of calcium-dependent genes, neuronal activity, and dendritic arborization in these neurons (Li et al., 2014).

On the other hand, treatment with paxilline, both in isolated RAW264.7 macrophage nuclei and whole cells, resulted in a dose-dependent increase in the phosphorylation of CREB in the nucleus (Selezneva et al., 2021). We recall here that treatment of the nucleus with high concentrations of Ca^2+^ also causes CREB phosphorylation. These results do not exclude a role for the BK channels located in other cell membranes, due to the high membrane permeability of paxilline, which would allow it to block the BK channels of both the plasma membrane and intracellular organelles (Selezneva et al., 2021).

## Function

### Mitochondrial BK Function

The functions of the mitoBK channel can be easily studied using isolated mitochondria. However, we cannot apply these studies’ results directly to intact cells (Li and Gao, 2016). It has been hypothesized that the activity of this channel is essential for mitochondrial function and homeostasis. mitoBK is expressed in IMM, in which they could regulate ion and protein movement involved cell apoptosis and the electron transport chain (ETC), respectively (Szabo and Zoratti, 2014; Li and Gao, 2016). Most studies have mainly focused on the cytoprotective effect on cardiac and neuro ischemia of mitoBK channels. Still, they have also shown significant evidence regarding mitochondrial structure and function, reactive oxygen species (ROS) regulation, mitochondrial Ca^2+^ retention capacity, and permeability transition pore (mPTP) activation in cellular respiration and cancer as well (see Figure 1A).

### MitoBK Channels in Cardioprotection

So far, the physiological role of mitoBK has been reported mainly by pharmacology or using genetic models (Szabo and Zoratti, 2014). Most studies have primarily focused on the cytoprotective effect on cardiac ischemia and reperfusion (I/R) injuries, to which mitoBK has been associated after the pioneering work of Xu et al. (2002). Using pharmacological agents to open and block the channel, mitoBK shows to be involved in such cardioprotection (Wang et al., 2004; Stowe et al., 2006; Bentzen et al., 2009, 2010; Borchert et al., 2013; Singh et al., 2013; Testai et al., 2013; Schmitt et al., 2014).

The cardioprotective effect mediated by the mitoBK channel is attributed mainly to (a) an increase in K^+^ in the mitochondrial matrix, (b) the retention of Ca^2+^, (c) a decrease in ROS, and (d) closure of the mPTP (Hermann et al., 2015). The flux of K^+^ from the cytosol to the negatively charged mitochondrial matrix is caused by the opening of mitoBK channels, which depolarizes the organelle (Szewczyk et al., 2006). The opening of mitoBK reduces the influx of Ca^2+^, decreasing the Ca^2+^ overload in the mitochondria (Xu et al., 2002; Du et al., 2020). Therefore, the functional effect of mitoBK channel activators is to reduce ROS production and Ca^2+^ overload, improving homeostasis and mitochondrial redox status after I/R as seen in isolated guinea pig hearts (Heinen et al., 2007a,b; Bentzen et al., 2009).

Due to nonspecific effects of drugs, the role of mitoBK in protection against I/R injury has been questioned, invoking biochemical and molecular reasons (see Gaspar et al., 2009; Szewczyk et al., 2009; Wojtovich et al., 2011, 2013). Conclusive evidence for the role of mitoBK in cardioprotection comes from studies using BK knockout mouse models (Kcnma1 −/−). The hearts of these mice are not protected from ischemic injury under treatment with NS1619 or NS11021. This lack of protection is revealed by measurements of cardiac function and infarct size in isolated perfused hearts (Singh et al., 2013; Wojtovich et al., 2013; Soltysinska et al., 2014). These experiments demonstrate that BK activator-mediated cardioprotection requires KCNMA1 expression and that mitoBK activation protects cardiomyocytes from ischemia and reperfusion injury (Singh et al., 2013; Goswami et al., 2019). Nonetheless, in vascular smooth muscle myocytes, the evidence suggests that mitoBK channels are not involved in protection against I/R injury (Frankenreiter et al., 2017).

### MitoBK Channel in Neuroprotection

Strong evidence shows that mitoBK channels located in IMM in neurons are associated with neuroprotective effects (Kulawiak and Szewczyk, 2012; Singh et al., 2013; Wojtovich et al., 2013; Soltysinska et al., 2014; Li and Gao, 2016; Krabbendam et al., 2018; Du et al., 2020). Kulawiak et al. (2008) demonstrated that opening of mitoBK located in IMM of rat brain, stimulated by CGS7184 and NS1619, inhibits hydrogen peroxide production by 20%. This effect is sensitive to BK channel blockers iberiotoxin and charybdotoxin. These results suggest that the opening of mitoBK inhibits ROS, promoting neuronal survival and neuroprotection (Kulawiak et al., 2008). However, Gaspar et al. (2009) proposed that the protective effect of NS1619 may not be mediated by mitoBK in every system. They studied primary rat cortical neurons and found that preconditioning with NS1619 caused mitochondrial depolarization, an effect that was displayed even with preincubation with paxilline (Gaspar et al., 2009). A possible explanation for this negative result could be that paxilline takes longer to diffuse through the plasma membrane and reach mitoBK in IMM (Balderas et al., 2015).

Subsequently, NS11021 was used to evaluate its cytoprotective effect on primary cortical neurons of rats with glutamate-induced excitotoxicity. On the one hand, due to the suppression of glutamate excitotoxicity, attenuation of oxidative stress, and preservation of mitochondrial function, mitoBK-dependent neuroprotection is induced (Borchert et al., 2011). On the other hand, the mitochondrial division inhibitor, mdivi-1, exhibited protective effects in ischemic injury by regulating the activation of mitoBK in mitochondria of cardiac neurons due to an increase in BK channel expression levels and attenuation of oxidative stress, mitochondrial dysfunction, and neuronal apoptosis (Liu et al., 2012). This neuroprotective effect is associated with the increase of mitochondrial Ca^2+^ and the decrease in ROS production mediated by mitoBK (Kulawiak and Szewczyk, 2012; Krabbendam et al., 2018; Du et al., 2020).

### MitoBK Channel in Mitochondrial Structure and Function

Opening of mitoBK channels has been found to regulate the respiratory rate, mitochondrial depolarization, matrix volume, and ROS production (Heinen et al., 2007a,b; Kulawiak et al., 2008; Hermann et al., 2015).

In muscle mitochondria from *Drosophila* mutants slo1- / - severe defects were found in terms of the mitochondrial ultrastructure, aberrations in the arrangement of ridges, an increased size (swollen) of the organelle, and loss of continuity of IMM compared to wild type cells expressing mitoBK (Gaspar et al., 2009). Meanwhile, Du et al. (2020) analyzed mitochondria of HEK and PC12 cells transfected with mutant BK channels (BKG354S, mutation that affects the selectivity filter). This mutation caused a selective loss of BK channels in the mitochondrial membrane and the loss of mitochondrial content, ranging from the loss of voltage-gated anion channel (VDAC) proteins to a reduction in every component mitochondrial oxidative phosphorylation (OXPHOS). This led to depolarized and dysfunctional mitochondria and the loss of the cytoprotective effect due to the activation of mitoBK (Du et al., 2020). Therefore, the mitoBK channel plays a crucial role in maintaining mitochondrial structure, function, and content.

That said, it has been found that mitoBK present in IMM contributes to the regulation of volume of the mitochondrial matrix, influences uptake of K^+^, mitochondrial transmembrane potential, pH balance, Ca^2+^ transportation, ROS production, mitochondrial dynamics in general and it has also been proposed to participate in increasing bioenergetic efficiency (Aon et al., 2010; Leanza et al., 2019). These may be considered as the mechanisms proposed for the cytoprotection above (Testai et al., 2015; Wawrzkiewicz-Jałowiecka et al., 2020).

### MitoBK Channel and ROS Regulation

The cardio- and neuroprotection conferred by mitoBK activators appears to be associated with the modulation of the rate of mitochondrial reactive oxygen species (ROS) generation in brain and heart cells (Andrukhiv et al., 2006; Facundo et al., 2006; Heinen et al., 2007a; Kulawiak et al., 2008; Krabbendam et al., 2018; Kshatri et al., 2018; Gururaja et al., 2019). The conclusive evidence of the key role of mitoBK channels in ROS generation comes from the use of genetic models, which demonstrated that the absence of BK channels increases ROS production. However, Soltysinska et al. (2014) reported that the knockout of mitoBK channels increased postanoxic ROS production in ventricular mitochondrial cells. This result strongly suggests that mitoBK channels regulate the production of ROS, as well as the oxidative state in hypoxia and reoxygenation of mitochondria. Moreover, Gururaja et al. (2019) found that in *Drosophila* mitoplasts that genetically blocking mitoBK channels increases ROS production, the consumption of O_2_ and the respiratory rate (Gururaja et al., 2019).

There is no consensus in the literature regarding the effect of mitoBK in the ROS production. Several reports demonstrate that MitoBK activation after I/R injury causes a reduction in ROS levels. ROS production increases when channel blockers are applied (Szewczyk et al., 2009; Cordeiro et al., 2015; Goswami et al., 2019). On the contrary, the activation of mitoBK in isolated and I/R injury-induced ventricular myocytes with NS11021, caused an increase in ROS levels. Addition of antioxidants, which decrease the open probability of mitoBK, abolished the increase in ROS production (Borchert et al., 2013). This increase in ROS production after mitoBK activation was also observed in a liver cancer cell line (Booth et al., 2016).

Ambivalence in responses after mitoBK activation could be related to some coupling between the channel and ROS generation sites. In the case that mitoBK is coupled to the mitochondrial complex I (reverse electron flow), the production of ROS should decrease upon mitoBK activation. However, if the channel is coupled to the mitochondrial complex III (direct electron flow), activation of mitoBK should lead to an increase in ROS (Krabbendam et al., 2018). In this regard, Stowe et al. (2006) showed in isolated mitochondria from cardiac cells that the succinate and rotenone-dependent H_2_O_2_ production that blocked reverse electron flow increased slightly after the activation of the mitoBK channel. On the other hand, Heinen et al. (2007a,b) demonstrated that in the absence of rotenone, under substrate conditions that allow reverse electron flow, mitoBK activation reduces H_2_O_2_ production by 73% by accelerating forward electron flow.

We note here that hemin, a by-product of hemoglobin with oxidative properties, can inhibit the electrical activity of BK channels. Therefore, the mitoBK channel can be considered a redox sensor. (Augustynek et al., 2014). Moreover, mitochondria of ventricular muscle fibers lacking mitoBK channels (by knockout) showed an increase in the production of postanoxic ROS, indicating that these channels regulate the oxidative state in hypoxia and reoxygenation (Soltysinska et al., 2014).

### MitoBK Channel and Mitochondrial Ca^2+^ Retention Capacity and mPTP Activation

MitoBK channel regulation of mitochondrial Ca^2+^ retention capacity could be observed pharmacologically activating the channel with NS1619, increasing the number of Ca^2+^ pulses necessary to cause a massive release of Ca^2+^ from the mitochondria (Singh et al., 2013). Ca^2+^ retention capacity in mitochondria is closely related to mPTP activation, which mediates Ca^2+^ release from mitochondria to the cytosol (Singh et al., 2013). In fact, in a study with rat liver, mitoplasts, and astrocytes, hypoxia inhibited mPTP but substantially increased mitoBK activity, with an increase in Ca^2+^ retention capacity, which was reduced using iberiotoxin (Cheng et al., 2008). This finding may suggest a functional link between mitoBK and mPTP, where the reduction of the activity of the mitoBK channel by mitochondrial substrates can support the activation of mPTP, leading to cell death by apoptosis (Laskowski et al., 2016).

Possibly by the opening of mPTP, apoptosis results from the complex interaction between Ca^2+^ and ROS. The activation of mitoBK is involved in both processes, linking this channel to a delay in the formation and/or closure of mPTP (Goswami et al., 2019). In single-channel recordings in rat astrocyte mitoplasts and hepatic mitochondria, inhibition of mitoBK channels by the pro-apoptotic protein BAX (B-cell lymphoma (Blc) -2-associated X) was observed, which in turn activated mPTP and induced cytochrome C release (an effect like that obtained using iberiotoxin (Cheng et al., 2008)). Conversely, BCL-Xl (an anti-apoptotic protein) inhibited the impact of BAX on mitoBK and mPTP blockade. mitoBK channel is related to apoptotic mechanisms mediated by BAX, which exerts its pro-apoptotic effect by inhibiting mitoBK and thus promotes the opening of mPTP (Cheng et al., 2008, 2011).

### MitoBK in Cellular Respiration

In the mitochondria of the human glioblastoma cell line U-87 MG, the substrates of the ETC (NADH, succinate, and malate or glutamate) and artificial donors of electrons (tetramethyl-p-phenylenediamine TMPD/ascorbate) inhibited the mitoBK channel (Bednarczyk et al., 2013b). These results suggest that the mitoBK channel is regulated by the cytochrome C oxidase and that a redox signal is “transferred” from ETC to mitoBK through CCO (Ohya et al., 2005). Together, these observations suggest a structural and functional coupling of the respiratory chain and mitoBK channels, although the underlying molecular mechanisms are still unknown (Laskowski et al., 2016).

Cytoprotection induced by mitoBK activators may also be mediated by inhibiting the mitochondrial respiratory chain (Kicinska and Szewczyk, 2004). Activating the mitoBK channel sing NS1619 in IMM of isolated rat brain mitochondria inhibited ROS production of the respiratory chain using the complex I (Kulawiak et al., 2008). In cultures of hippocampal sections exposed to glutamate, preincubation with NS1619 showed an increase in basal respiration (Piwońska et al., 2016). Activating cardiac mitoBK channels produced an improvement in mitochondrial respiration due to a decrease in state 4 respiration (characterized as a state without any ATP usage/production), while state 3 of respiration (described as a state with saturating ATP usage/production) was unchanged (Aon et al., 2010). These findings suggest a probable mitoBK-dependent mechanism for both cardiac and neuronal cytoprotection (Testai et al., 2015; Wawrzkiewicz-Jałowiecka et al., 2020).

### MitoBK and Cancer

To date, a possible role for mitoBK in cancer development has not been reported; despite that, it has been related to the survival and motility of glioma cells after irradiation (Steinle et al., 2011). Irradiation and hypoxia (Gu et al., 2014) have been found to increase the P_o_ of mitoBK, which in turn activates Calmodulin kinase II (CaMKII), leading to increased migration of glioblastoma cells (Steinle et al., 2011; Peruzzo et al., 2016), as well as resistance to hypoxic conditions (Gu et al., 2014). MitoBK in gliomas may also regulate the respiratory chain and confer cytoprotection, which may be one reason that makes this type of cancer incurable (Wawrzkiewicz-Jałowiecka et al., 2020).

### MitoBK and Kidney Transplantation

Shrum et al. (2019) demonstrated that mitoBK channels might represent a therapeutic target to prevent cold storage (CS) preservation and rewarming (RW)-induced kidney injury that is very common in kidneys routinely subjected to transplant. To do this, they added NS11021 to the CS solution and evaluated the effect on normal rat kidney proximal tubular epithelial cells. The addition of this activator of mitoBK prevented the deterioration induced by CS+RW in the uptake of K^+^ mediated by mitoBK, as well as a reduction in cell death and mitochondrial damage. In addition, they observed mitigation in respiratory dysfunction, depolarization, and superoxide production (Shrum et al., 2019).

### Nuclear BK Function

Even though the presence of BK channels has been reported in the NE of many cell types such as pancreatic cells, brain endothelial cells and macrophages (Maruyama et al., 1995; Gobeil et al., 2002; Selezneva et al., 2021), little is known about its functional role.

### nBK and Nucleoplasmic Ca^2+^ Signaling

In pancreatic acinar cell nuclei, nBK channels only localize in ONM, and their activation is sensitive to Ca^2+^ lumen levels (Maruyama et al., 1995). Whether nBK can regulate the nuclear transmembrane potential was proven in mice hippocampal neurons using a potentiometric probe. This experiment showed that the perinuclear lumen got more negative when nBK was blocked by paxilline. Usage of paxilline also indicated an increase in nuclear Ca^2+^ through RyR, mainly due to intracellular BK inhibition without pmBK being involved (Li et al., 2014). We recall here that RyR is sensitive to changes in nuclear transmembrane potential (Zahradníková and Mészáros, 1998). This increase in nuclear Ca^2+^ due to nBK inhibition showed to activate CREB through phosphorylation in a nuclear Ca^2+/^CaMKIV-dependent manner, which also causes changes in neuronal dendritic arborization (Li et al., 2014).

Not only nBK but also pmBK are found in macrophages from the nervous system (microglia). These channels are involved in pro-inflammatory mechanisms induced by Toll-like receptors 4 (TLR4) activated by lipopolysaccharides (LPS). pmBK is activated through TLR4, which induces translocation of NF-kB (nuclear factor kappa B) to the nucleus, where it prompts gene expression regarding cytokine production. Treatment with paxilline at different times after LPS application showed that after 6h, paxilline did not affect cytokine production, indicating the existence of BK modulation on gene expression NF-kB-independent. This result becomes clear by considering that the nBK expression is induced by LPS long-lasting activation on TLR4 (Yang et al., 2019). Another signaling mechanism in macrophages regarding nBK was described using the RAW264.7 cell line. The blockage of nBK using paxilline in preparations of the cell line and isolated nuclei showed an increase in CREB phosphorylation due to CaMKII Calmodulin kinase II) and CaMIV (Calmodulin kinase IV) activity (see Figure 1B; Selezneva et al., 2021). It is important to note that CREB is related to many roles for macrophages, particularly preventing apoptosis (Park et al., 2005).

## Discussion and Concluding Remarks

Compared to the vast information available for pmBK, one may get the impression that there is a lack of evidence for mitoBK and nBK. Nonetheless, there has been an increase in studies regarding their biophysical and pharmacological properties during recent years (Ohya et al., 2005; Li et al., 2014) and how these studies relate to their functional role in different cell types (Gobeil et al., 2002; Selezneva et al., 2021; Wawrzkiewicz-Jałowiecka et al., 2021).

mitoBK is sensitive to multiple stimuli regarding mitochondrial function (like Ca^2+^, membrane potential and O_2_). It also regulates ETC, ROS production, and apoptosis (Siemen et al., 1999; Heinen et al., 2007a,b; Cheng et al., 2008; Kulawiak et al., 2008; Hermann et al., 2015). On the other hand, nBK plays a role in nuclear Ca^2+^ signaling and induction of gene expression under the effect of different drugs (Yang et al., 2019; Selezneva et al., 2021). However, there is a lack of research regarding nBK biophysical properties and how these can determine the underlying mechanisms (Li et al., 2014).

Both in nucleus and mitochondria membranes K^+^ flow is essential to maintain ionic homeostasis and hence a myriad of cell functions. The electrochemical driving force for ion movement across membranes varies in different intracellular organelles. In case of the nucleus, K^+^ concentration is higher than in the cytoplasm, while in mitochondria it is lower, which causes a large influx of K^+^ toward the perinuclear space and into the mitochondrial matrix, respectively. Even though other potassium channels are expressed in both the mitochondrial and nuclear membranes, BK channels are high conductance, where a single BK channel can transport up to 10^8^ ions per second, generating a significant change in K^+^ flux and, therefore, changes in membrane potential in the different organelles (Singh et al., 2012). Thus, mitoBKs as well as the other mitochondrial K^+^ channels participate in the mitochondrial K^+^ cycle, which consists in a balance between the electrophoretic uptake of K^+^ in the mitochondrial matrix and the diffusive leakage of this ion, mediated by the K^+^/H^+^ exchanger (Garlid and Paucek, 2003; Szabo et al., 2012; Schulz and Di Lisa, 2016).

As we mentioned the pmBK channels regulate membrane potential, ionic homeostasis, calcium signaling, and cell volume (Latorre et al., 2017). Functions that are also reported for mitoBK and nBK in the mitochondria and nucleus, respectively. For this reason, it would be expected that the pharmacological or genetic modulation of these channels would serve as therapeutic targets. Pharmacological and genetic activation of mitoBK results in cellular and organic protection against I/R injury, giving this channel a promising therapeutic approach as a potential target in the treatment of cardiovascular and neurodegenerative diseases, as well as a potential drug target in organ transplant and cancer medicine (Singh et al., 2012; Laskowski et al., 2016; Leanza et al., 2019). Conversely, nBK represents a new strategy to develop effective therapies in neurodegenerative diseases such as Alzheimer and autism (Li et al., 2014). However, despite the obvious structural and functional importance of the nucleus, nuclear ion channels, their characteristics, and potential therapeutic targets remain largely unknown.

It is unfortunate that exclusive modulators of mitoBK have not yet been reported and the low selectivity and pleiotropic effects of its agonists have hindered the development of a treatment that exclusively involves the activation of mitoBK (Gururaja et al., 2019). The molecular identification of the regulatory and pore-forming subunits of mitoBK channels would provide more possibilities for the development of therapeutic strategies based on the selective modulation of mitoBK in various tissues (Wrzosek et al., 2020).

Overall, the study of BK role in double-membrane organelles such as mitochondria and nucleus is in the need of a more detailed research regarding the differences between organelle BK channels and pmBK concerning their biophysics and pharmacological properties. This knowledge can determine the still unknown molecular mechanisms involving their functional role in their respective organelles and how they can work as possible targets in different pathological conditions.

## Author Contributions

All authors contributed to the writing, revising, and approval of the manuscript equally.

## Funding

This work is supported by FONDECYT Grants 1190203 (to RL), The Centro Interdisciplinario de Neurociencia de Valparaíso (CINV) is a Millennium Institute supported by the Millennium Scientific Initiative of the Chilean Ministry of Economy, Development and Tourism. ANID doctorado nacional 21200592 fellowship (to NG-S). ANID doctorado nacional 21202097 fellowship (to FE).

## Conflict of Interest

The authors declare that the research was conducted in the absence of any commercial or financial relationships that could be construed as a potential conflict of interest.

## Publisher’s Note

All claims expressed in this article are solely those of the authors and do not necessarily represent those of their affiliated organizations, or those of the publisher, the editors and the reviewers. Any product that may be evaluated in this article, or claim that may be made by its manufacturer, is not guaranteed or endorsed by the publisher.

## Abbreviations

Aβ: Amyloid β-sheet fibrils
BAX: Bcl-2-associated X
Bcl-XL: B-cell lymphoma-extra-large
BK: Large-conductance calcium- and voltage-activated potassium channel
CaMKII: Calmodulin kinase II
CCO: Cytochrome C oxidase
CGS718: Ethyl2-hydroxy-1-[[(4-methylphenyl)amino]oxo]-6-trifluoromethyl-1H-indole-3-carboxylate
CGS7184: Ethyl 1-[[(4-chlorophenyl)amino]oxo]-2-hydroxy-6-trifluoromethyl-1H-indole-3-carboxylate
ChTX: Charybdotoxin
CREB: cAMP response element-binding protein
CS: Cold storage
diCl-DHAA: 12,14-dichloro dehydroabietic acid
eNOS: Endothelial nitric oxide synthase
EP_3_: Perinuclear prostaglandin receptors
ETC: Electron transport chain
H_2_O_2_: Hydrogen peroxide
I/R: Ischemia and reperfusion injuries
IbTX: Iberiotoxin
IMM: Inner mitochondrial membrane
INM: Inner nuclear membrane
InsP3R: Inositol-1,4,5 trisphosphate (InsP3) receptors
Kv: K+voltage-dependent channels
LPS: Lipopolysaccharides
mdivi-1: Mitochondrial division inhibitor
mitoBK: Mitochondrial BK
mPTP: Mitochondrial permeability transition pore
nBK: Nuclear BK
NE: Nuclear envelope
NF-kB: Nuclear factor kappa B
NPo: Absolute open probability
ONM: Outer nuclear membrane
PD: Pore domain
pmBK: BK channel in the plasma membrane
*P_0_*: Open probability
RCK: K+−conductance regulators
ROS: Reactive oxygen species
RW: Rewarming
RyR: Ryanodine receptors
S0-S6: Transmembrane segments
TLR4: Toll-like receptors 4
TMPD: Tetramethyl-p-phenylenediamine
VSD: Voltage sensor domain
γ: Unitary conductance
ΔΨ: Electrochemical membrane potential
